# *In vitro* characterization and genome sequencing of two novel lytic phages against *Salmonella* Infantis isolated from poultry feces

**DOI:** 10.3389/fmicb.2024.1479700

**Published:** 2024-12-05

**Authors:** Noemi Battistelli, Fabrizia Tittarelli, Fausto Ruffini, Luigi Gavazzi, Silvia Scattolini, Vicdalia Aniela Acciari, Teresa Romualdi, Valentina Curini, Simona Di Carlo, Nicola D’Alterio, Giacomo Migliorati, Francesco Pomilio, Giuseppe Aprea

**Affiliations:** ^1^Experimental Zooprophylactic Institute of Abruzzo and Molise “G. Caporale”, Teramo, Italy; ^2^Gesco Cons Coop arl, Cesena, Italy

**Keywords:** antimicrobial agents, bacteriophages, genome sequencing, poultry, *Salmonella* Infantis

## Abstract

**Introduction:**

*Salmonella* spp. is the second most common bacteria associated with foodborne gastrointestinal outbreaks in humans, with the highest contamination levels in meat, especially poultry. *Salmonella enterica* subsp. *enterica* serovar Infantis is the primary serovar isolated from broilers, without causing any symptomatic disease. Conversely, certain human strains can result in symptomatic illness (fever, headache, and diarrhoea). Therefore, reducing *S. Infantis* colonization in broilers is important before slaughter, to prevent this pathogen carryover along the food chain.

**Methods:**

Here, we report the characterization of two *S. Infantis* virulent phages, isolated from broiler feces. Isolates were phenotypically and genetically characterized.

**Results and discussion:**

Phages (ɸ) SaI_NFG_5581 and SaI_NFG_5577 were characterized as strictly lytic versus *S. Infantis* but with different bacteriolytic activities and genetic features. They both belong to the Caudoviricetes class, but ɸSaI_NFG_5581 (genome length 112,970 bp) belongs to the Demerecviridae family while ɸSaI_NFG_5577 (genome length 42,481 bp) to the Guernseyvirinae family. Genomic analysis excluded the presence of lysogeny, toxin, or antimicrobial resistance genes, and for those reasons, the two phages could be considered safe. Phages are stable under a broad range of pH (4-10) and temperature (4°C-50°C) conditions. *In vitro*, both ɸSaI_NFG_5581 and ɸSaI_NFG_5577 were able to lower *Salmonella* counts of about 2.2 LOG/mL and 3.4 LOG CFU/mL respectively, at MOI 0.1 after 2 h of treatment. After 24 h, *Salmonella* counts treated with both phages remained lower than the control (non-phage-treated *Salmonella*). These newly isolated phages have promising features, which could be exploited and further studied for potential *in vivo* application.

## 1 Introduction

*Salmonella* spp. is the second most common bacteria, after *Campylobacter* spp., associated with foodborne gastrointestinal outbreaks in humans in the European Union (EU). It has been one of the leading causes of foodborne epidemics in EU member states and third countries, as reported in the 2023 zoonosis report by the European Food Safety Authority (European Food Safety Authority and the European Centre for Disease Prevention and Control, 2023a), with a total of 65,208 reported cases of human salmonellosis in 2022 (European Food Safety Authority and the European Centre for Disease Prevention and Control, 2023a). The percentage of hospitalized cases was 38.9%, slightly higher than that of 2021, with a mortality rate per case in the EU of 0.22%, similar to that of 2021. *Salmonella enterica* subsp. *enterica* serovar Infantis ranks among the top five *Salmonella* serovar acquired in the EU involved in human infections, with a 2.3% presence, following *S. enteritidis* and *S. typhimurium* (European Food Safety Authority and the European Centre for Disease Prevention and Control, 2023a).

According to European Parliament Regulation (EC) (2003)/No. 2160 and subsequent amendments, Member States in Europe must establish National control programs (NCP) for *Salmonella* (NSCP), aimed at reducing, at the farm level, the prevalence of those serovars considered relevant to public health. Moreover, they should ensure that proper and effective measures are taken, particularly at primary production levels. Member States should also verify the compliance with process hygiene criteria on carcasses at the slaughterhouse, in the context of European Parliament Regulation (EC) (2005)/No 2073, which lays down microbiological criteria, intended as food safety criteria and process hygiene criteria, for *Salmonella* in specific food categories (European Food Safety Authority and the European Centre for Disease Prevention and Control, 2023a).

Despite strict measures carried out by NCSP, the persistence of multidrug-resistant (MDR) serovars, such as *S. Infantis*, in broiler chickens has emerged as a significant threat to food safety (Vandeplas et al., 2010; European Food Safety Authority and European Centre for Disease Prevention and Control, 2022). In 2022, the highest percentages of *Salmonella*-positive samples were found in turkeys (14%), broiler chickens (11.8%), pigs (2.7%), cattle (0.96%), and sheep (0.75%) (European Food Safety Authority and the European Centre for Disease Prevention and Control, 2023a).

Among *Salmonella* serovars, *S. Infantis* has been considered the number one for isolation in broiler chicken production in many EU Member States since 2014 (European Food Safety Authority and the European Centre for Disease Prevention and Control, 2023a).

This serovar may possess various genetic strategies (e.g., presence of antimicrobial resistance genes, heavy metal resistance, mobile virulence genes, biofilm formation capacity) that could enhance their epidemiological fitness and provide additional virulence and persistence traits throughout the food chain (Alba et al., 2023). However, a significant issue is the high persistence of multidrug-resistant (MDR) serovars, such as *S. Infantis*, in broiler chickens, which currently represent one of the main threats to food safety. Multidrug-resistant (MDR) *S. Infantis* strains in broilers, turkeys, and laying hens reached 83.9, 88.8, and 24.5%, respectively. Furthermore, 38% of *S. Infantis* strains isolated from human clinical samples were found to be MDR; they were resistant to eight out of nine antimicrobial classes tested and susceptible only to meropenem (European Food Safety Authority and the European Centre for Disease Prevention and Control, 2023b). The persistence and resistance of *Salmonella* are also often linked to its ability to form biofilms and resistance to disinfectants (Steenackers et al., 2012; Corcoran et al., 2014). *Salmonella* spp. can diffuse to humans through the farm-to-fork process, especially when consuming contaminated food of animal origin (Teklemariam et al., 2023). To control these food-borne infections, it is essential to implement preventive measures at every stage of the food chain. To date, none of the treatments adopted (i.e., vaccination and feed additives) are fully effective in eliminating *Salmonella* contamination from poultry flocks. As a result, the most efficient strategy for reducing the prevalence of this pathogen at the farm level appears to be the implementation of a combination of several measures (Vandeplas et al., 2010).

Therefore, the reduction of *S. Infantis* colonization in broilers before slaughter, and surveillance of emerging *Salmonella* serovars, are important actions to be seriously addressed, especially in those cases where the bacteria have developed mechanisms to survive even in challenging environmental conditions (Chousalkar and Willson, 2022).

Bacteriophages (phages) are viruses that can infect and kill bacteria without negative effects on any eukaryotic cell. Literature analysis has shown that the rapid increase in MDR bacteria worldwide, coupled with a decline in the development and production of new chemical antibiotics, and the efficacy of bacteriophages also against AMR bacterial strains, is leading scientists to reconsider them for the treatment of bacterial infections (Karn et al., 2023). Characterized by a very narrow spectrum of activity, phages avoid the most significant problem closely associated with antibiotic administration, e.g., the impact on the entire microbiome with the elimination of potentially beneficial bacteria, the excessive growth of secondary pathogens, and the emergence of resistant bacteria (Domingo-Calap and Delgado-Martínez, 2018).

Moreover, phages can be also used in combination with antibiotics, to treat bacterial infections (Principi et al., 2019). In fact, previous works experienced their synergistic effect (phage-antibiotic synergy - PAS) which involves an increase in phage virulence due to the administration of sublethal concentrations of antibiotics (Li et al., 2021).

Bacteriophages have the advantage of being significantly safer and well tolerated, as they replicate only in the target bacterium (host) but cannot infect mammalian cells. This conclusion seems to be supported by all past experiences gathered in Eastern Countries (i.e., Georgia) and by all studies conducted more recently in humans (Kakasis and Panitsa, 2019) and experimental animals (Hall et al., 2012), which have not reported significant adverse effects following bacteriophage administrations (for a Review see Fang et al., 2024). Currently, in the EU, phage therapy is admitted only in the veterinary sector (European Parliament EU Regulation, 2019/6; EMA/CVMP/NTWP/32862/2022); moreover, the European Medicines Agency (EMA) has recently opened a public consultation on a “concept paper on the development and manufacture guidelines for human medicinal products tailored to phage therapy” [EMA/CHMP/BWP/486838/2023; European Medicines Agency (EMA), 2023].

With the restriction or elimination of antibiotic use in food animals, researchers have been looking into the use of bacteriophages to manage food-borne infections. Bacteriophages might be able to operate as a last line of defense when antibiotics are either not available or inefficient. Phages could represent a cost-effective and innovative alternative in poultry farms, especially against MDR strains (for a review see Abd-El Wahab et al., 2023).

For all those premises, more data on phage characterization are certainly necessary to gain new insights and to broaden the knowledge for a safe and efficient application in therapy.

This study aimed to isolate and characterize specific lytic phages against *S. Infantis* from poultry farms where the incidence of this pathogen is high to use phage therapy for reducing *S. Infantis* loads in poultry at the time of slaughtering, though mitigating the spread along the food chain.

## 2 Materials and methods

### 2.1 Sample collection

Poultry feces were collected from 8 broiler farms situated in Abruzzo and Molise regions (Central Italy), between February 2023 and February 2024. Twenty samples from each farm were collected from different boxes, put in sterile bags, and delivered to the laboratory the same day (*n* total samples = 100). Farms were selected because of the high prevalence of *S. Infantis*.

### 2.2 Bacterial strains (hosts) and culture conditions

Strains of *S. Infantis* were used in this study as phage-hosts. The strains belonged to the bacteria culture collection of Istituto Zooprofilattico Sperimentale dell’Abruzzo e del Molise (IZS-Teramo, Italy). The strains (2023.18489.1; 2023.18489.2; 2023.18489.3; 2023.18489.4; 2023.18489.5) were isolated from poultry farms in 2021–2022, according to ISO 6579-1:2017/Amd 1:2020. All strains were grown in Luria-Bertani (LB) broth (Oxoid™, Hampshire, United Kingdom) at 37°C for 16–24 h.

### 2.3 Phage isolation and plaque purification

Phage isolation was performed with two methods, direct isolation and isolation after enrichment.

For direct isolation, 5 g of feces were homogenized 1:10 with LB broth containing 1 M CaCl_2_ and 1 M MgSO_4_ and stored overnight at 4°C. The following day, samples were centrifuged at 5,000 *g* for 10 min and filtered through 0.45 μm filters (Merck - Darmstadt, Germany).

Isolation after enrichment consisted of homogenization of 5 g of feces with LB 5X broth containing 1 M CaCl_2_, 1 M MgSO_4,_ and 500 μL of a mixture of 5 *S. Infantis* strains (2023.18489.1; 2023.18489.2; 2023.18489.3; 2023.18489.4; 2023.18489.5) available in our strains collection. Samples were incubated overnight at 37°C with shaking at 180 rpm and filtered through 0.45 μm filters, after the incubation time.

The filtrates from both direct isolation and enrichment treatments were employed for phage isolation by the double-layer agar technique as described by Scattolini et al. (2021), with some modifications. One hundred microliters of each host, 100 μL of 1 M CaCl_2,_ and 100 μL of 1 M MgSO_4_ were inoculated in 4 mL of LB soft agar (LB broth +0.7% w/v agar). The solution was vortexed and poured onto an LB agar plate (LB broth +1.5% w/v agar). Plates were left to dry at room temperature for about 20 min. Ten microliters of sample filtrates were spotted on the plates and incubated overnight at 37°C. The plates were observed for the presence of transparent areas or plaques at the inoculation point. The zones of lysis, where present, were collected by cutting them out from the agar with a sterile loop and put in 3 mL of SM broth (50 mM Tris–HCl pH7.5, 100 mM NaCl, 8 mM MgSO4, 0.01% Gelatin (v/w)) and left to elute for 8 h at room temperature. Phage suspensions were then filtered through 0.45 μm filters, and incubated overnight at 37°C, in liquid LB media, with the host used for isolation, to let phages replicate and increase the titer (phage propagation). After incubation, the suspension was centrifuged at 5,000 *g* for 10 min and filtered through 0.45 μm filters. The filtered suspension was serially diluted 1:10 until 10^−8^ and plaque assay was performed. A single plaque was then collected by cutting it out from the agar with a sterile loop, put in SM broth and left to elute for 8 h at room temperature. Phage suspension was then filtered and propagated in liquid media as described before. This whole protocol was repeated at least three times until plaques did not have the same morphology (phage purification). Purified phages were titred through plaque assay, according to Van Charante et al. (2021).

### 2.4 Host range and specificity assessment

Host range was assessed using spotting methods as described by Sun et al. (2022). A total of 42 strains (19 *S. Infantis*, 9 *S. typhimurium*, 10 *Escherichia coli*, 2 *Campylobacter jejuni*, and 2 *C. coli*) were used to determine the host range and assess phage specificity. All strains belonged to the bacteria culture collection of IZS-Teramo. A scale from 0 to 3 was used to determine the lysis zones: 0 refers to no lysis, and 3 indicates a fully degraded bacterial lawn, as reported by Molina et al. (2020).

### 2.5 One-step growth curve

One-step growth curve (OSGC) was determined as reported by Rivera et al. (2022), with minor modifications. Briefly, the host strain was grown at 37°C to mid-exponential phase (OD_600nm_ = 0.3; approx. 1 × 10^7^ CFU/mL) in 20 mL of LB medium. Phages were added to achieve Multiplicity of Infection (MOI) 0.1 (10^6^ PFU/mL). The mixture was then incubated at 37°C by shaking at 125 rpm for 15 min, to allow phages to absorb to the cells, and then the mixture was centrifuged at 7000 *g* for 5 min to separate free phages from the sample. The supernatant was discarded and then the 20 mL of pre-warmed LB was quickly added. The phage was immediately diluted and plated for phage titer assay at T0. The suspension was incubated with shaking at 125 rpm and the aliquots were collected at 10 min intervals for 60 min, filtered through 0.45 μm syringe filters, and immediately diluted and plated for phage titer assays, using the agar overlay method. The burst size was calculated through the quantification of the infective centers (average three higher viral titers/three lower viral titers), while the latency period was calculated as the mean between these time points (immediately post-lysis) and the previous time point (immediately pre-lysis) (Rivera et al., 2022). Results are expressed as the mean of three replicates ± standard deviation.

### 2.6 pH and thermal stability assays

Thermal stability was examined following Imklin and Nasanit (2020) with some modifications. SM buffer was pre-incubated at 4, 25, 37, 50, and 70°C for 10 min, to reach the desired temperatures. Phages (10^7^ PFU/mL) were inoculated and incubated at specific temperatures for 1 h. After treatment, phage titration was performed with agar overlay assay as described before.

The stability of phages at different pH was evaluated by inoculating 10^7^ PFU/mL of phage in 1 mL of SM buffer previously adjusted with 1 M NaOH or 1 M HCl to reach a pH range from 2 to 10. Phage preparations were incubated at room temperature for 60 min (Zhao et al., 2019).

Results are expressed as PFU/mL. Each assay was performed in triplicate and the results are expressed as the means of the three replicates ± standard deviation.

### 2.7 Characterization of the bacteriolytic activity

The bacteriolytic activity was determined by plating methods (Mulani et al., 2022). Briefly, phage suspension of each phage was mixed with mid-log-phase *S. Infantis* (10^7^ CFU/mL) at different MOIs (ratio of phages to bacteria): 0.01, 0.1, 1, 10, and 100 in 20 mL of LB medium. The mixtures were incubated at 37°C after adding 200 μL of 1 M CaCl_2_ and 200 μL of 1 M MgSO_4_ to each tube. One mL of each sample was taken every two hours for six hours, and after 24 h, and separated into two fractions. A fraction was ten-fold serially diluted, spread on LB plates, and incubated at 37°C overnight, for bacterial cell count, expressed as LOG CFU/mL. Another fraction was firstly centrifuged at 7000 *g* for 5 min and then filtered through a 0.45 μm syringe filter. The lysate was overlaid on the LB plates and incubated at 37°C overnight. Plaques were counted and the phage titer was calculated as LOG PFU/mL. After checking the best MOI, the bacteriolytic activity of both phages together was tested. Phages were inoculated at a MOI of 0.1 both sequentially and together (1:1 proportion) and incubated at 37°C after adding 200 μL of 1 M CaCl_2_ and 200 μL of 1 M MgSO_4_. For sequential inoculation, ɸSaI_NFG_5577 was inoculated first and ɸSaI_NFG_5581 was added after 3 h. For simultaneous inoculation (cocktail), both the phages were added together. One mL of each sample was taken every 2 h for 6 h, and after 24 h, Samples were ten-fold serially diluted, spread on LB plates, and incubated at 37°C overnight, for bacterial cell count, expressed as LOG CFU/mL.

### 2.8 DNA extraction

To extract DNA, phages were concentrated to obtain a high titer of pure phage stocks. Phage initial titers were 4×10^8^ PFU/mL for ɸSaI_NFG_5581 and 1×10^9^ PFU/mL for ɸSaI_NFG_5577. Concentration was carried out by ultrafiltration with Amicon® Ultra-15 centrifugal filter units Ultracell 100 kDa membrane (Merck - Darmstadt, Germany) following Bonilla and Barr (2018) protocol, with substantial modifications. Five mL of ≈ 10^8^ PFU/mL of phage lysate were added into the upper reservoir of the Amicon® filtration device and centrifuged at 4000 *g* for 15 min. Using a pipette, the filter was washed with 1 mL of phosphate-buffered saline (PBS) solution (137 mM NaCl, 2.7 mM KCl, 10 mM Na_2_HPO_4_, and 1.8 mM KH_2_PO_4_) and phage lysate was collected from the upper reservoir. Samples were successively filtered and centrifuged again with the Amicon® filtration device at 4000 *g* for 15 min. The filter was washed with 400 μL of PBS and phage lysate was collected from the upper reservoir. One hundred microliters were used to asses phage titer and the remaining volume was treated with DNase and RNase to remove any residual bacterial DNA and RNA from the hosts. One hundred microliters DNase I 10 x buffer, 10 μL DNase I (20 U) (New England Biolabs, Ipswich, MA, United States) and 2 μL RNase A (10 mg/mL) (Merck) were added to 1 mL of sample and incubated for 1.5 h at 37°C without shaking. After incubation, 40 μL of 0.5 M EDTA (Invitrogen, Life Technologies, New York, United States) was added to inactivate DNase I and RNase A (Jakočiūnė and Moodley, 2018). One hundred microliters were used to assess phage titers.

The final phage concentration employed for DNA extraction was 1×10^9^ PFU/mL for ɸSaI_NFG_5581 and 7×10^9^ PFU/mL for ɸSaI_NFG_5577. DNA extraction was performed with High Pure Viral Nucleic Acid Kit (Roche - Mannheim, Germany) following the manufacturer’s instructions. Total DNA was quantified with Qubit DNA HS assay (Thermo Fisher Scientific Inc., Waltham, MA, United States).

### 2.9 Phage genome sequencing and sequence analysis

Whole genome sequence analysis (WGS) was performed for ɸSaI_NFG_5581 and ɸSaI_NFG_5577. The libraries were constructed using Illumina® DNA Prep (M) Tagmentation (96 Samples) (Illumina Inc., San Diego, CA, United States), according to the manufacturer’s protocol. Deep sequencing was performed on the NextSeq500 platform (Illumina Inc., San Diego, CA, United States) using the NextSeq 500/550 Mid Output Reagent Cartridge v2 (300 cycle) (Illumina Inc., San Diego, CA, United States) and standard 150 bp paired-end reads. After obtaining the raw reads, the most suitable tools for phages were chosen (for a review, see Shen and Millard, 2021). On the GENPAT platform,^1^ after a quality check and trimming of raw reads data using FastQC v0.11.5 and Trimmomatic v0.36 (Bolger et al., 2014), respectively host depletion was performed by Bowtie2 (Langmead and Salzberg, 2012) using accession number NZ CP016406.1 as reference.

The *de novo* assembly of paired-end reads (Assembler Geneious), and normalization of the reads (BBNorm, v38.84, Bushnell et al., 2017) were performed using the software Geneious Prime® (v2024.0.4).

The whole genome sequence identities were mapped against other nucleotide sequences using the Blast program of the National Center for Biotechnology Information (NCBI). The most similar phage genomes were downloaded from the NCBI database to assess the average nucleotide identity and to compute a phylogenetic analysis. The phylogenetic tree was created with the Geneious Prime® (v2024.0.4) program using the Neighbor-Joining method, with 1,000 bootstrap replicates. The sequences used for the tree were the following: *Enterobacteria* phage Chi (NC021315), *Escherichia* phage BB1 (MT843274), *Escherichia* phage ECOP18 (MT080103), *Escherichia* phage EscoHU1 (LC659915), *Escherichia* phage phiX174 (NC001422), phage vB_Sen_I1 (MT233524), *Salmonella* phage vB_SenTO17 (MT012729), *Salmonella* phage Spc35 (NC015269), *Salmonella* phage vB_SalS_SA001 (MN445182), *Salmonella* phage 118970_sal1 (NC031930), *Salmonella* phage 100268_sal2 (NC031902), *Salmonella* phage 9NA (NC025443), *Salmonella* phage vB_SenS_SB9 (MK867835), *Salmonella* phage S131 (NC048009), *Salmonella* phage vB_Sen-E22 (MT311645), *Salmonella* phage vB_SenS_SE1 (MK479295), *Salmonella* phage PSDA-2 (MW725301), *Salmonella* phage TS6 (NC069148), *Salmonella* phage BP12C (NC031228), *Salmonella* phage FSL SP-030 (NC021779), *Salmonella* phage Chi (NC025442), *Salmonella* phage GRNsp27 (NC069147), *Salmonella* phage St162 (NC069146), *Salmonella* phage Shemara (NC069152), *Salmonella* phage FSL SP-031 (NC021775), *Salmonella* virus VSiA (MN393079), and *Salmonella* virus VSiP (NC069153).

Coding sequence annotations were evaluated by using a rapid, free-to-use, and consistent genomic annotation tool dedicated to bacteriophages: Pharokka software (v1.7.1) (Bouras et al., 2023) using PHANOTATE database. The resulting output, a. gff file, was employed to construct the genome annotation maps using Geneious Prime®. tRNA genes were detected using the tRNAscanSE program (Lowe and Chan, 2016). The DNA polymerase sequences of the phages and other homologous sequences were retrieved from the GenBank database. Geneious Prime® was used to construct the phylogenetic tree, with the Neighbor-Joining method, and 1,000 bootstrap replicates. Genomic comparisons were made with Progressive MAUVE alignment (Darling et al., 2010). The complete genome sequences of the two *Salmonella* phages have been submitted to GenBank (ɸSaI_NFG_5581: PQ488695; ɸSaI_NFG_5577: PQ488694).

### 2.10 Statistical analysis

All experiments were conducted as three independent tests. The results were expressed as mean values ± standard deviation (SD). The statistical significance of the data was determined by two-way ANOVA analysis of variance using GraphPad Prism version 8.0.0 for Windows (GraphPad Software, Boston, Massachusetts, United States). A *p*-value less than 0.05 was considered statistically significant.

## 3 Results

### 3.1 Phage isolation, host range and plaque morphology

**Figure 1:**
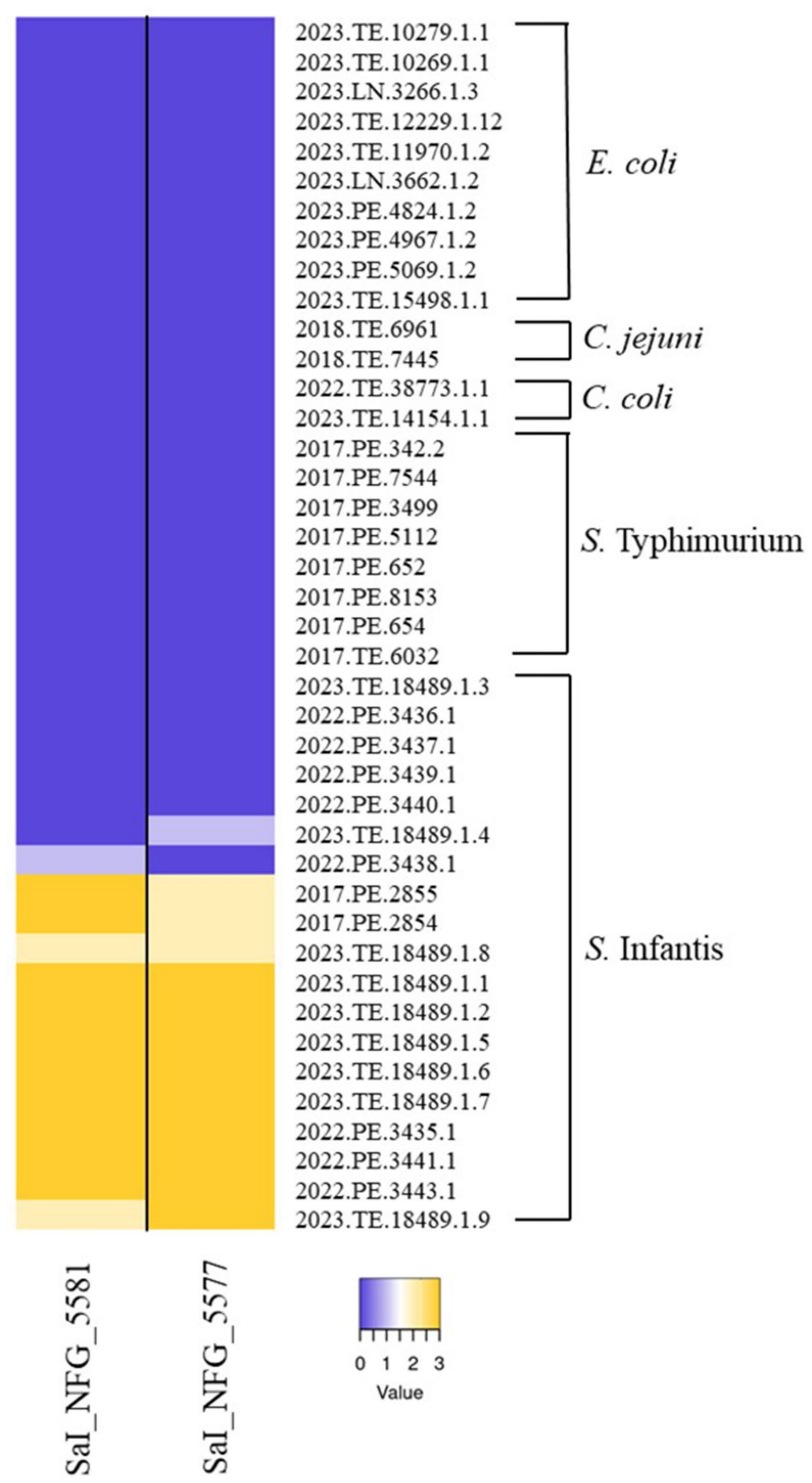
Host range of isolated phages. Heatmap represents the lysis profiles of phages versus different strains of *S. Infantis*, *S.* Typhimurium, *E. coli*, *C. jejuni,* and *C. coli*. Values range from 0 (Dark purple, no lysis) to 3 (Dark yellow, complete lysis).

A total of 40 *Salmonella* phages were isolated from 100 poultry feces samples collected from 8 Italian broiler farms. All phages were strictly active versus *S. Infantis* strains, with some differences in terms of efficacy (data not shown). Two phages, ɸSaI_NFG_5581 and ɸSaI_NFG_5577, were chosen for further characterization. Figure 1 shows the host range of the two phages against 19 strains of *S. Infantis*, 8 strains of *S.* Typhimurium, 10 strains of *E. coli*, 2 strains of *C. jejuni,* and 2 strains of *C. coli*. Light yellow and dark yellow refers to almost complete or complete lysis. Phages showed lytic activity versus 12 *S. Infantis* strains and none or poor lysis (Dark and Light purple) against the other 7 strains of *S. Infantis*. ɸSaI_NFG_5581 and ɸSaI_NFG_5577 were not able to form plaques against all the strains belonging to the other species employed.

Phages, ɸSaI_NFG_5581 and ɸSaI_NFG_5577 have different plaque morphologies. Phage SaI_NFG_5581 plaques have a 1 mm diameter while ɸSaI_NFG_5577 are slightly bigger, with a diameter of 3 mm. Both have a halo surrounding the center of the plaque (Supplementary Figure S1).

### 3.2 *In vitro* characterization of ɸSaI_NFG_5581 and ɸSaI_NFG_5577

#### 3.2.1 Determination of the burst size

**Figure 2:**
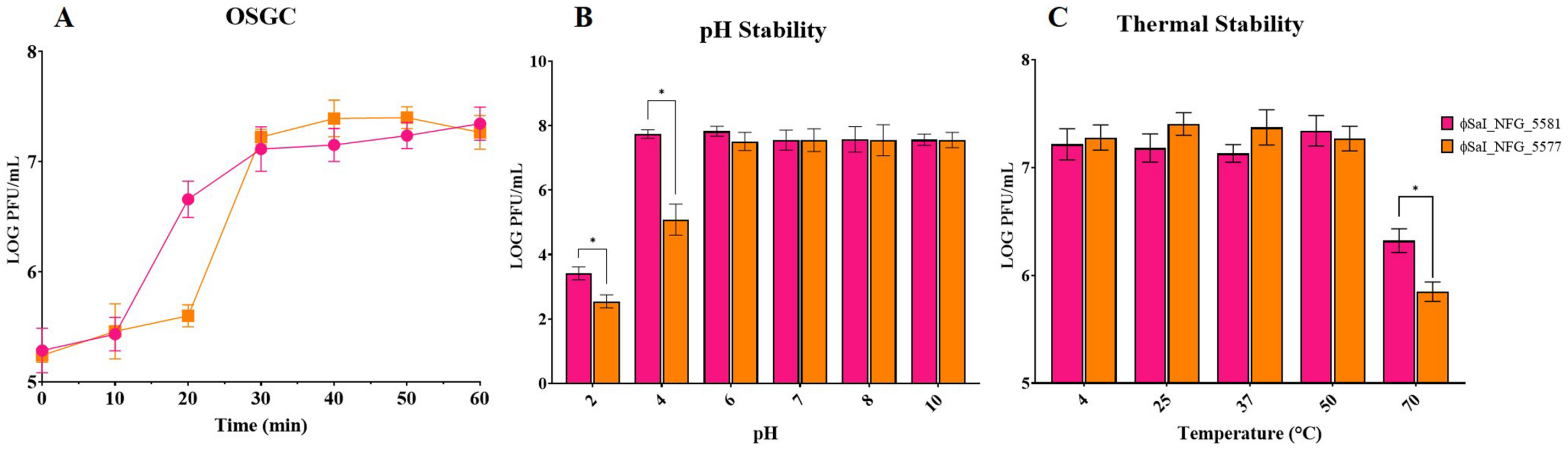
*In vitro* characterization of phages: burst size, pH and thermal stability. **(A)** One-step growth curve of ɸSaI_NFG_5581 and ɸSaI_NFG_5577. Phage titers (PFU/mL) were assessed for 1 h, at 10 min interval; **(B)** pH stability of ɸSaI_NFG_5581 and ɸSaI_NFG_5577 at different pH (2, 4, 6, 7, 8, 10) for 1 h; **(C)** thermal stability of ɸSaI_NFG_5581 and ɸSaI_NFG_5577 at different temperatures (4°C, 25°C, 37°C, 50°C and 70°C) for 1 h. Values are expressed as the mean of three replicates ± SD. Significance levels are depicted as *p < 0.01 calculated by ANOVA with Dunnett’s test.

The OSGC assay of ɸSaI_NFG_5581, grown under standard conditions using LB medium, with a MOI of 0.1, showed a latency period of 10 min and a burst size of 22 ± 5 viral particles, while ɸSaI_NFG_5577 showed a latency period of 20 min and a burst size of 82 ± 10 viral particles (Figure 2A).

#### 3.2.2 pH and thermal stability

The two phages had a similar behavior also at different pH ranges. They were stable between pH 6 and 10, while titers dropped at pH 2. The titer of ɸSaI_NFG_5577 diminished at pH 4, reaching a value of 5 LOG PFU/mL (Figure 2B).

Phage viability at different thermal conditions was examined. Over an hour, phages were found to be stable between 4°C and 50°C. SaI_NFG_5581 and SaI_NFG_5577 phage titers similarly dropped by about 1 LOG PFU/mL at 70°C (Figure 2C).

#### 3.2.3 Bacteriolytic activity

**Figure 3:**
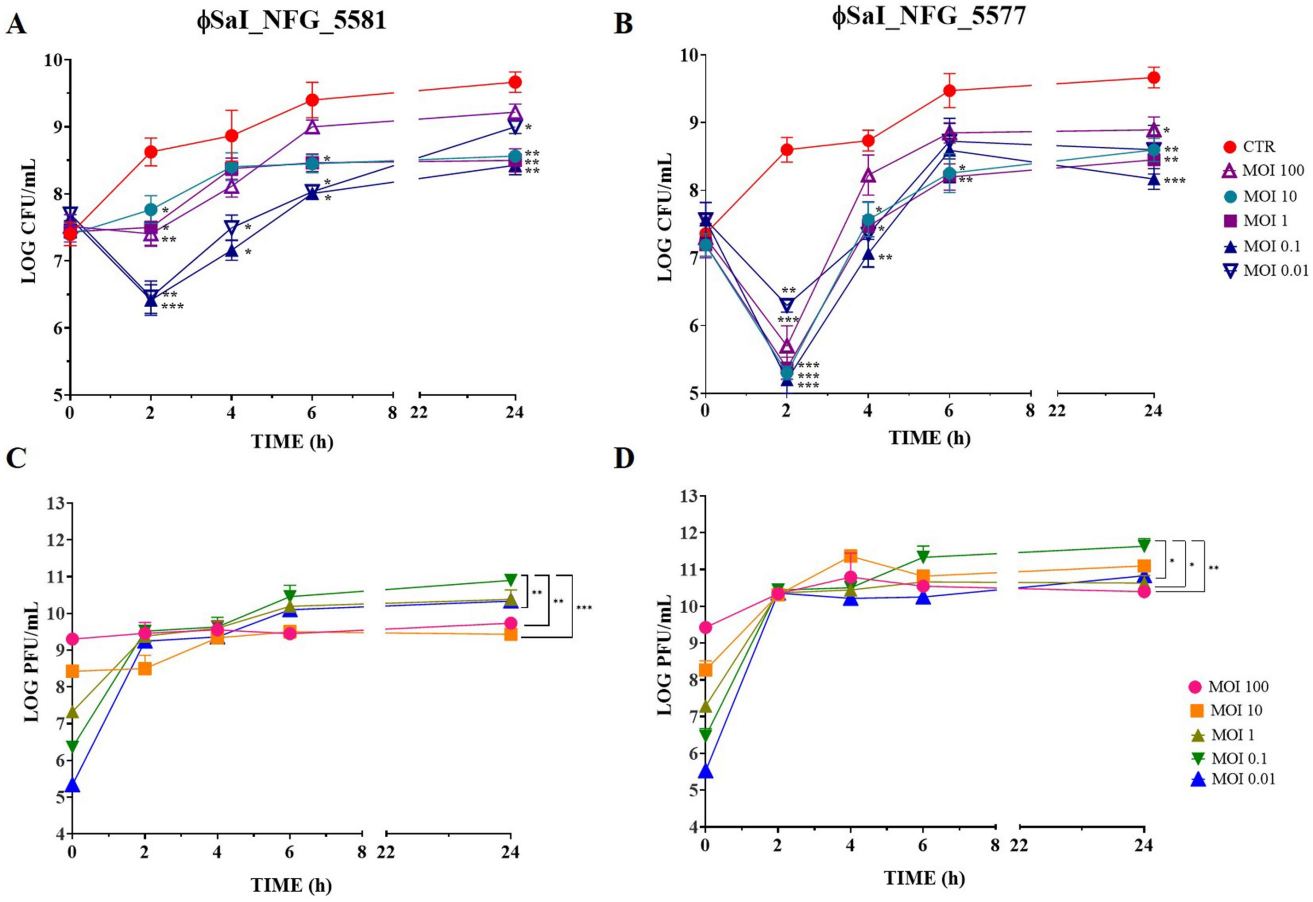
Bacteriolytic activity and replication of phages ɸSaI_NFG_5581 and ɸSaI_NFG_5577. **(A)** *Salmonella* viable counts at different MOI concentrations of phages (MOI 100, 10, 1, 0.1, and 0.01) at different time points (T0, T2, T4, T6, T24 hours); **(B)** *Salmonella* viable counts at different MOI concentrations (MOI 10, 1 and 0.1) at different time points (T0, T2, T4, T6, T24 hours); **(C)** ɸSaI_NFG_5581 titers at different MOI concentrations (MOI 100, 10, 1, 0.1 and 0.01) at different time points (T0, T2, T4, T6, T24 hours); **(D)** ɸSaI_NFG_5577 titers at different MOI concentrations (MOI 100, 10, 1, 0.1 and 0.01) at different time points (T0, T2, T4, T6, T24 hours). Values are expressed as the mean of three replicates ± SD. Significance levels are depicted as ***p < 0.0001; **p < 0.001; *p < 0.01 calculated by ANOVA with Dunnett’s test.

Results of *Salmonella* counts and phage titers at the different tested MOIs are reported in Figure 3. *Salmonella* viable counts without phages (CTR) reached about 9 LOG CFU/mL after 6 h and remained stable for up to 24 h; instead, *Salmonella* viable counts treated with both the phages, at the MOIs tested, were always kept at lower values than the control CTR. The best results were observed when *Salmonella* was treated with both phages at MOI 0.1. In particular, after treatment with ɸSaI_NFG_5581, *Salmonella* counts significantly dropped by 2.2 LOG CFU/mL after 2 h, compared to CTR (*p* < 0.001). The *Salmonella* counts increased both in CTR and with ɸSaI_NFG_5581 during 24 h observation, but a significant difference between CTR and *Salmonella* with ɸSaI_NFG_5581 of about 1.2 LOG CFU/mL was always kept up to 24 h (*p* < 0.01; Figure 3A). The same trend was observed also for *Salmonella* counts treated with ɸSaI_NFG_5577, but the difference observed at 2 h post phage addition was significatively wider, of about 3.4 LOG CFU/mL (*p* < 0.001; Figure 3B). In relation to phage titers, at MOI of 0.1, ɸSaI_NFG_5581 and ɸSaI_NFG_5577 reached values of 10.5 and 11 LOG PFU/mL after 6 h, respectively, which were slightly higher values than titers observed at MOI 100, 1 and 0.01 (Figures 3C,D). The titers remained almost stable for up to 24 h.

**Figure 4:**
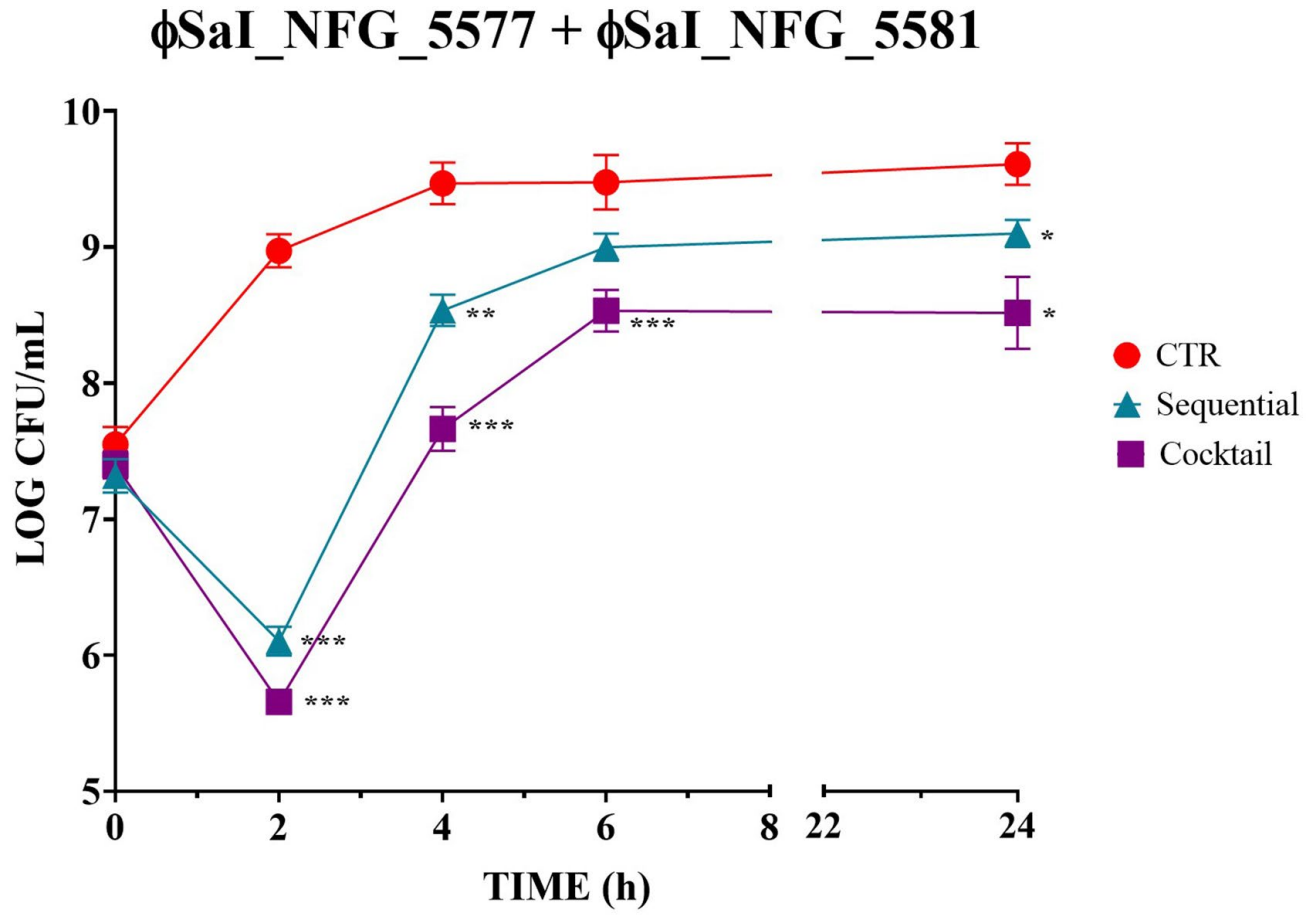
Bacteriolytic activity of phages ɸSaI_NFG_5577 and ɸSaI_NFG_5581 used in sequential or simultaneous inoculation (cocktail). *Salmonella* viable counts after treatment with phages at MOI 0.1 at different time points (T0, T2, T4, T6, T24 hours). For sequential inoculation, the second phage ɸ (SaI_NFG_5581) was inoculated after 3 h. For simultaneous inoculation (cocktail) phages were added together at time 0. Values are expressed as the mean of three replicates ± SD. Values are expressed as the mean of three replicates ± SD. Significance levels are depicted as ***p < 0.0001; **p < 0.001; *p < 0.01 calculated by ANOVA with Dunnett’s test.

Since the results pointed out that MOI 0.1 was the best to treat *Salmonella*, this value was employed to check whether the combination of phages could enhance bacteriolytic activity. Figure 4 shows *Salmonella* counts after sequential and simultaneous (cocktail) inoculation of phages. The cocktail of phages showed the best results in terms of efficacy, leading to significant drop of bacterial counts of about 2.5 LOG CFU/mL after 2 h (*p* < 0.0001) and of 1.1 LOG CFU/mL after 24 h (*p* < 0.01), compared to the untreated control. When phages were used in sequence, *Salmonella* viable counts dropped by 1.5 LOG CFU/mL after 2 h (*p* < 0.0001) and 0.1 LOG CFU/mL after 24 h (*p* < 0.01).

### 3.3 Genomic and phylogenetic characterization

**Figure 5:**
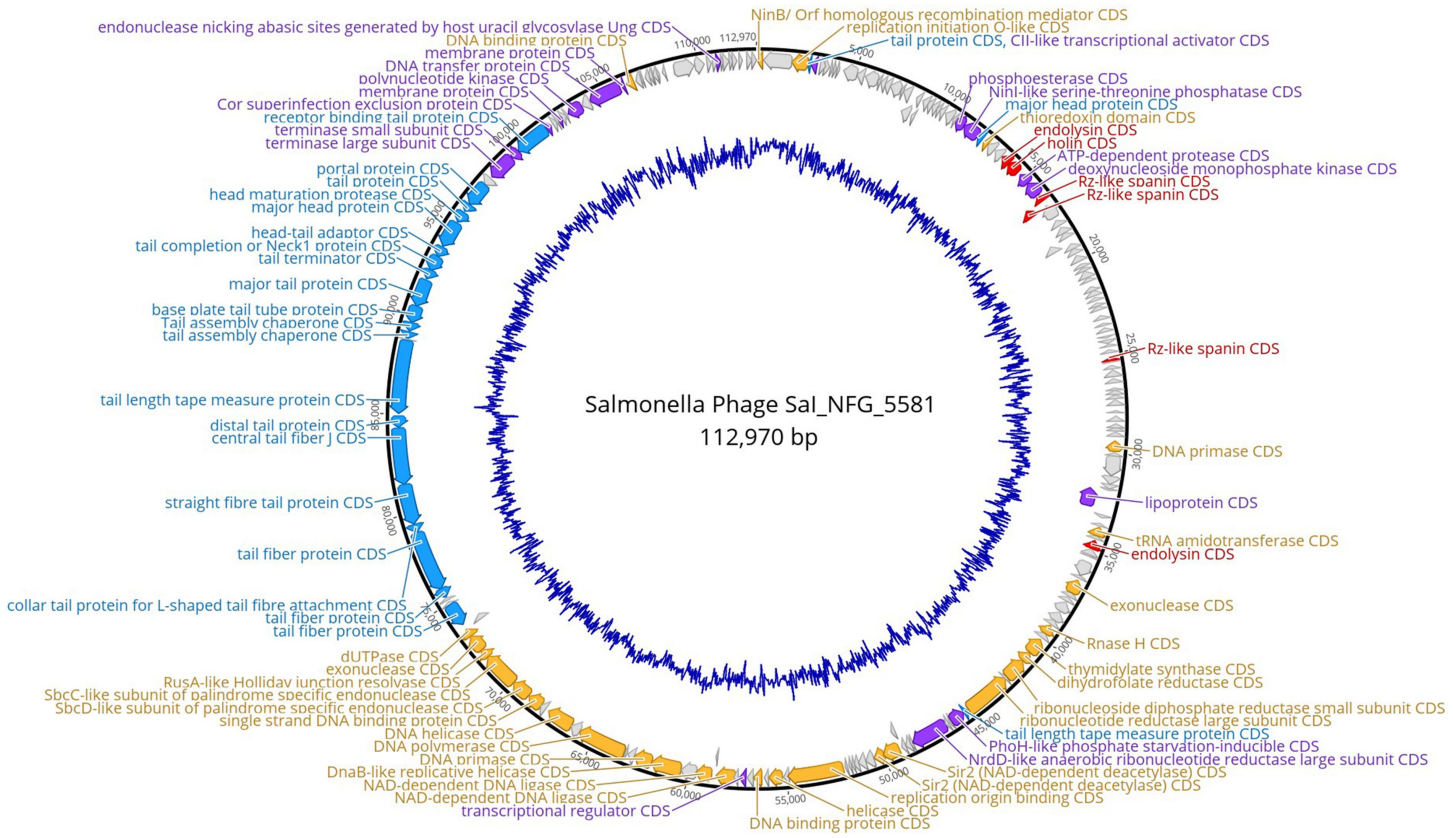
Genome map of phage ɸSaI_NFG_5581. The predicted CDSs are represented with different colors according to their function. Blue: structural proteins; orange: nucleotide synthesis proteins; red: lysis-related proteins; purple: other functional proteins; grey: hypothetical proteins. The innermost blue circle represents %G + C.

*Salmonella* phage ɸSaI_NFG_5581 genome revealed a length of 112,970 bp and G + C content of 39.4%. Phage SaI_NFG_5581 was found to be closely related to *Salmonella* phage Spc35, which is assigned to the lineage Duplodnaviria; Heunggongvirae; Uroviricota; Caudoviricetes; Demerecviridae; Markadamsvirinae; Tequintavirus; *Tequintavirus* SPC35 (Genbank accession number NC_015269), with an 84.1% identity. Next, a total of 209 CDSs were identified, 134 of which had a leftward orientation and 75 of which had a rightward orientation. Additionally, the majority of all CDSs can initiate translation with an AUG, 7 CDSs started with UUG, and 23 started with GUG. Phage SaI_NFG_5581 contained 2 different restriction endonucleases. Finally, 19 tRNA genes were detected in the genome sequence. The putative products predicted for each CDS are shown in Figure 5.

**Figure 6:**
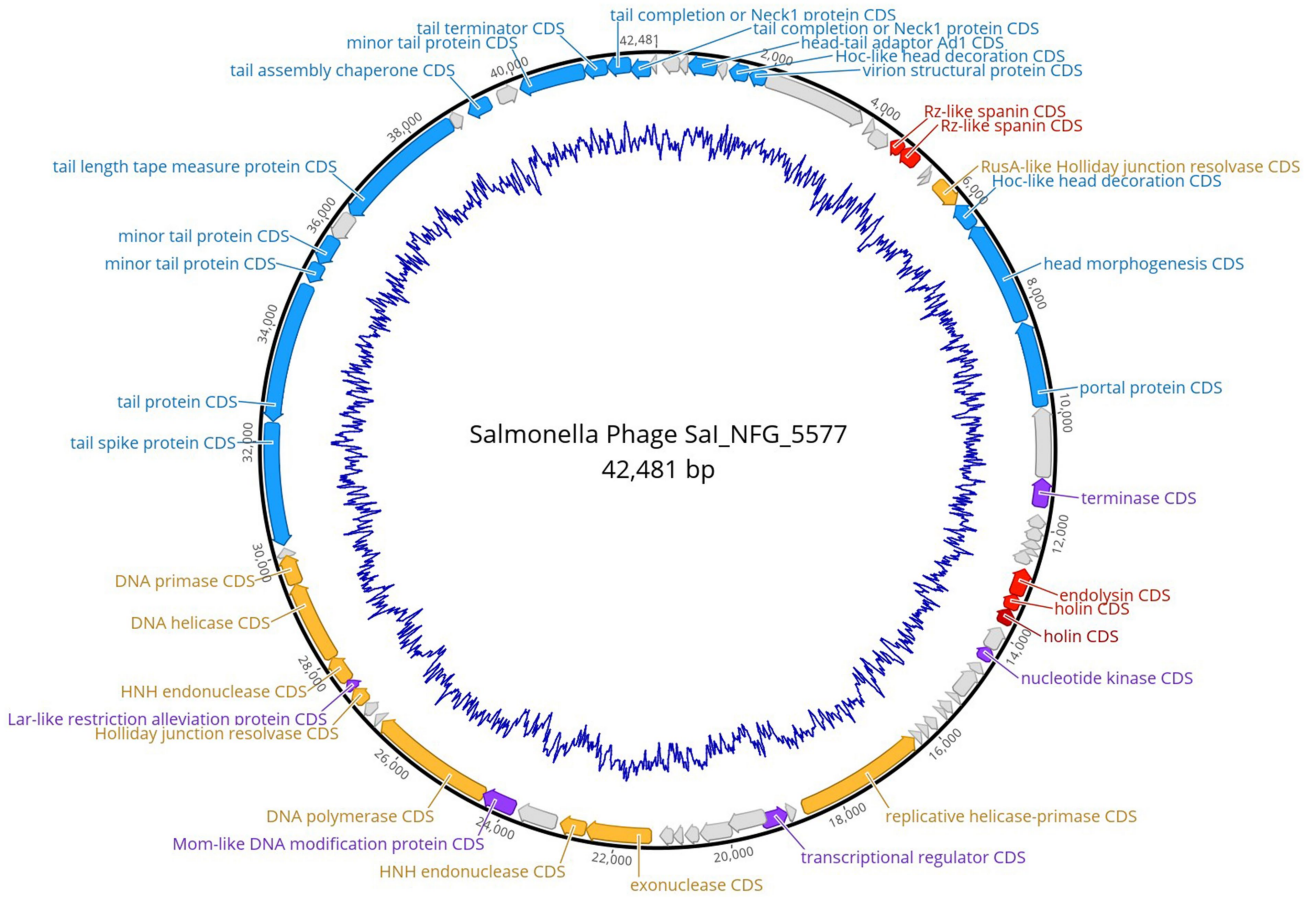
Genome map of phage ɸSaI_NFG_5577. The predicted CDSs are represented with different colors according to their function. Blue: structural proteins; orange: nucleotide synthesis proteins; red: lysis-related proteins; purple: other functional proteins; grey: hypothetical proteins. The innermost blue circle represents %G + C.

*Salmonella* phage ɸSaI_NFG_5577 genome had a length of 42,481 bp, and a G + C content of 51.3%. ɸSaI_NFG_5577 was found to be closely related to *Salmonella* phage vB_SenS_SE1, which is assigned to the lineage: Duplodnaviria; Heunggongvirae; Uroviricota; Caudoviricetes; Guernseyvirinae; Cornellvirus; *Cornellvirus* vB_SenS_SE1; (Genbank accession number NC021775), with a 73.53% identity. Next, a total of 72 CDSs were identified, 47 of which had a leftward orientation and 26 of which had a rightward orientation. Additionally, the majority of all CDSs can initiate translation with an AUG, 3 CDSs started with GUG, and only two started with UUG. Phage SaI_NFG_5577 contained 2 different restriction endonucleases. Finally, none of the tRNA genes was detected. The putative products predicted for each CDS are shown in Figure 6.

**Figure 7:**
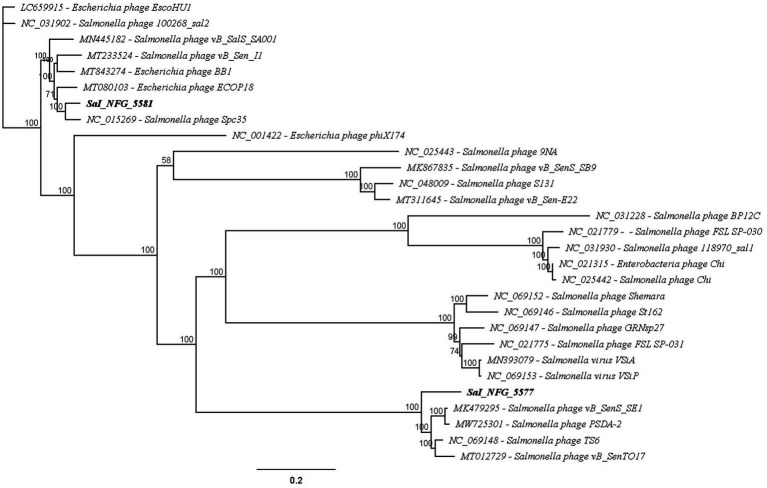
Phylogenetic analysis of WGS of isolates compared to known phages against *Salmonella* and *Escherichia* spp. Neighbor-Joining method and 1,000 bootstrap replicates were employed to build the tree.

The putative products of the CDSs were predicted according to the amino acid sequences and homology to functional domains aligned with known phages. They were categorized into five modules, including structural proteins (blue), such as tail fiber components/proteins and capsids and scaffold proteins; nucleotide synthesis proteins (orange), such as the coding DNA polymerase, DNA helicase, etc.; putative lytic enzymes (red), such as lysozyme, endolysin, holins etc.; other functional proteins (purple), and hypothetical proteins (grey), as shown in Figures 5, 6. No CDSs that are associated with lysogeny, human virulence genes (i.e., integrases or repressors), or drug resistance were found. Table 1 summarizes the main characteristics of ɸSaI_NFG_5581 and ɸSaI_NFG_5577. The phylogenetic analysis of the whole genome sequences is shown in Figure 7.

**Table 1:** Summary of the principal characteristics of ɸSaI_NFG_5581 and ɸSaI_NFG_5577.

	ɸSaI_NFG_5581	ɸSaI_NFG_5577
Host Specificity	*S. Infantis*	*S. Infantis*
Class	Caudoviricetes	Caudoviricetes
Family	Demerecviridae	Guernseyvirinae
Genus	Tequintavirus	Cornellvirus
Genome size	112,970 bp	42,481 bp
G + C percentage	39.4%	51.3%
Number of CDS	209	72
Plaque diameter	1 mm	3 mm
Burst size (viruses)	22 (± 5) viral particles	82 (± 10) viral particles
Latent period	10 min	20 min

**Figure 8:**
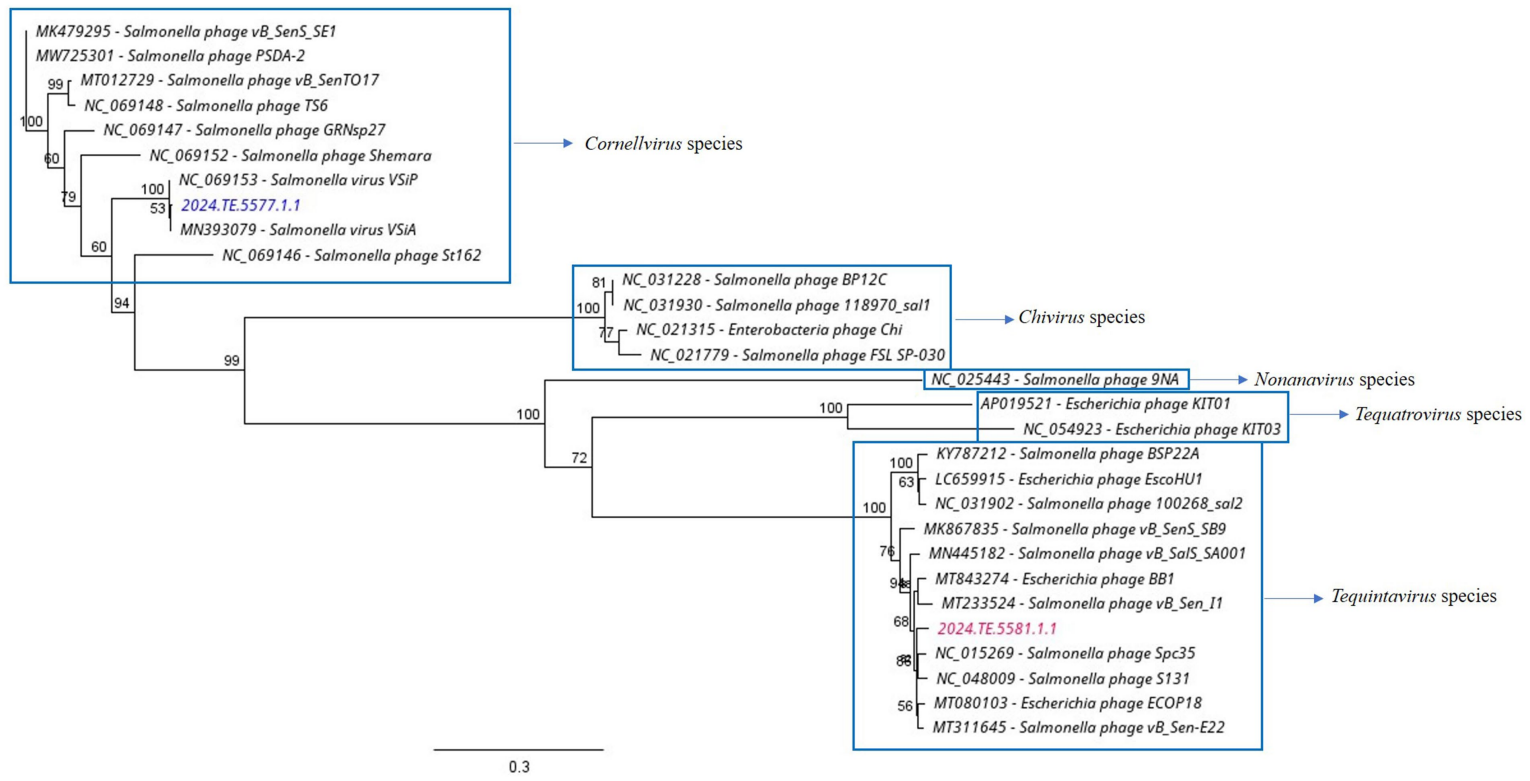
Phylogenetic tree of ɸSaI_NFG_5581 and ɸSaI_NFG_5577 based on DNA polymerase sequences.

**Figure 9:**
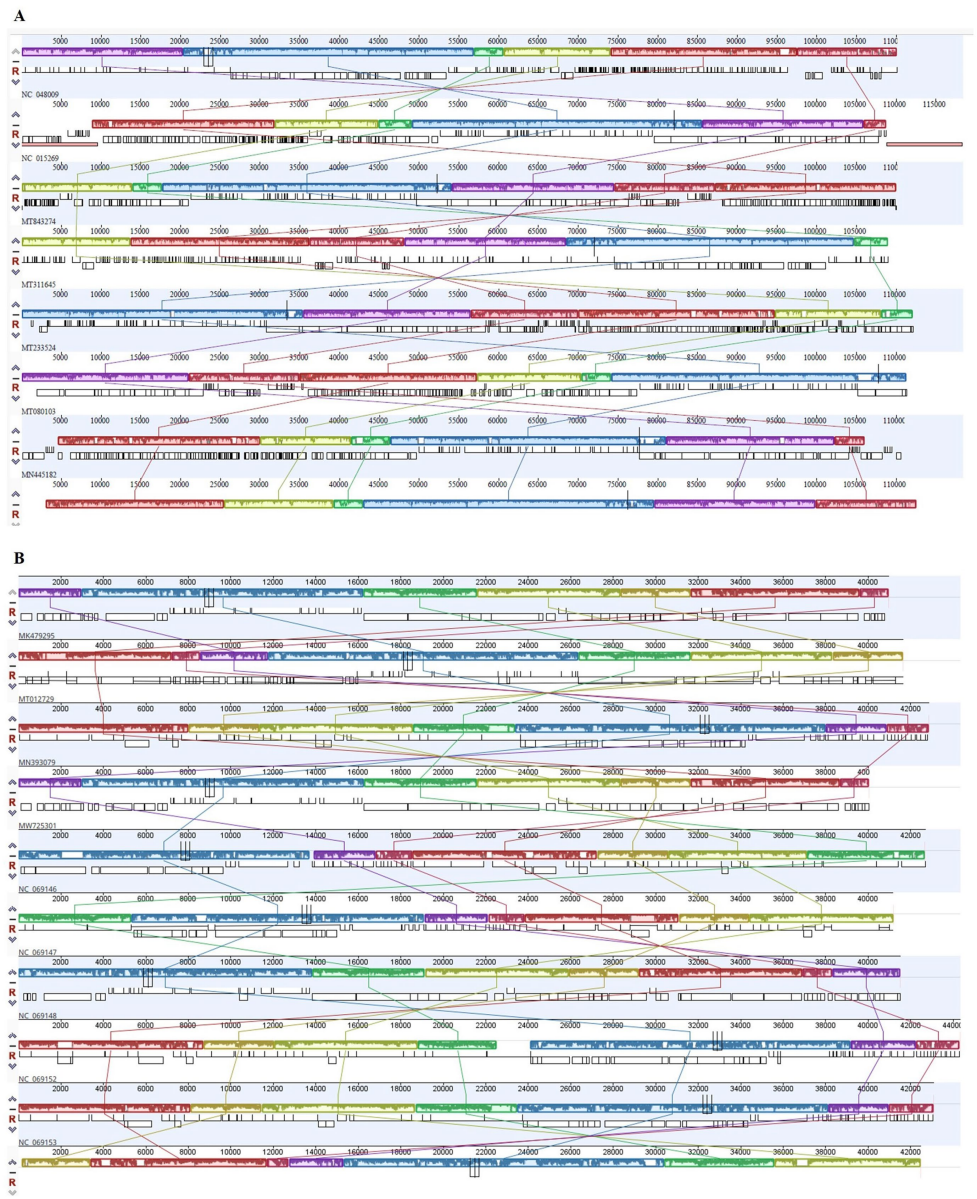
Progressive Mauve alignment of ɸSaI_NFG_5577 **(A)** and ɸSaI_NFG_5581 **(B)** with homologous phages genomes. Boxes with identical colors are homologous DNA regions.

The analysis demonstrated that ɸSaI_NFG_5581 belongs to the *Tequintavirus* species, while ɸSaI_NFG_5577 to the *Cornellvirus* species (Figure 8). Moreover, the genome similarities of ɸSaI_NFG_5577 with closely related strains (*Salmonella* phage vB_SenS_SE1, *Salmonella* phage vB_SenTO17, *Salmonella* virus VSiA, *Salmonella* phage PSDA-2, *Salmonella* phage St162, *Salmonella* phage GRNsp27, *Salmonella* phage TS6, *Salmonella* phage Shemara, *Salmonella* virus VSiP), and of ɸSaI_NFG_5581 with closely related strains (*Salmonella* phage S131, *Salmonella* phage Spc35, *Salmonella* phage BP12C, *Salmonella* phage vB_Sen-E22, *Salmonella* phage vB_Sen_I1, *Escherichia* phage ECOP18, *Salmonella* phage vB_SalS_SA001), were compared with progressive Mauve Alignment. The results showed that the gene sequences of ɸSaI_NFG_5581 and ɸSaI_NFG_5577 had a high degree of consistency with their respective relative phages, and all of these phages contain six locally collinear blocks (Figures 9A,B).

## 4 Discussion

*Salmonella* is the leading cause of foodborne illness related to the contamination of poultry meat and its products. Consuming food contaminated with *Salmonella* can result in bacterial food poisoning; therefore preventing *Salmonella* contamination in meat is crucial. Moreover, the constant emergence of bacterial resistance to antibiotics is one of the reasons why the research of alternative solutions has recently increased. Lytic phages are considered among the most promising antibacterial compounds to be used in animals and throughout meat processing and production, to reduce bacterial contaminations (Garvey, 2022; Alomari et al., 2021). Due to their high host specificity, the use of bacteriophages often requires the availability of a collection of many different phages. In the selection process of phages, it is essential to ensure that they do not carry genes for toxins or other harmful molecules for humans and animals. Moreover, phages that can rapidly amplify and lyse are preferred because they can quickly eliminate the target bacteria. Consequently, for effective application, a large variety of *Salmonella* bacteriophages must be isolated and characterized (Caflisch et al., 2019; Górski et al., 2020).

In this study, the authors initially isolated and identified 40 *Salmonella* phages from poultry feces samples. Two of them were selected, named ɸSaI_NFG_5581 and ɸSaI_NFG_5577, *in vitro* characterized and studied from a genetic point of view.

To observe the latent period and burst size of phages, a one-step growth curve (OSGC) was used. OSGC is an experimental curve that quantitatively describes the growth pattern of phages and it is considered an important assay to asses phages lytic activity (Rohde et al., 2018). The latent period found for ɸSaI_NFG_5581 in 10 min, while it is longer for ɸSaI_NFG_5577 (20 min). The burst size is 22 viral particles for ɸSaI_NFG_5581 and 82 viral particles for ɸSaI_NFG_5577. These data are similar to those reported by Abdelsattar et al. (2023) where ɸZCSE9 showed a latent period of 10 min and a burst size of 20 viral particles.

Phage SaI_NFG_5581, showed a lower burst size while ɸSaI_NFG_5577 showed a longer latent period, but a higher burst size, which can ensure effective removal of host bacteria, even if in a relatively longer time, in comparison to the other phage. These data reflect the different bacteriolytic activities of the two phages.

Stability assays are necessary to clarify the behavior and storage conditions of the phages intended to be used in practice. The authors found that both phages have favorable thermostability and pH stability. In addition, the pH and thermal stability of phages have to be assessed and taken into close consideration for future potential applications too (i.e., phage therapy). For pH stability, both phages remained at the same titer between pH 4–10 which makes them suitable for different applications, but there was a drop at pH of 2. Due to this behavior, some actions to protect them should be taken into account if used in environments where pH is lower or equal to 2. Both phages showed high thermal stability, up to 50°C, with a decrease of ±1 log PFU/mL at 70°C. These results are supported also by other authors; Lu et al. (2020) found that ɸvB_SenS_SE1 begins to lose potency when the temperature is greater than 50°C. Similar stability was found also by other authors while testing *Salmonella* phages (Pan et al., 2023; Abdelsattar et al., 2023; Khan et al., 2024). All phages survived at room temperature, which is an important parameter for phages considered to be used in therapy.

To assess whether the isolated phages can efficiently kill the target bacteria, their bacteriolytic activity against a specific host was explored. Tests were conducted at different MOIs, which is the parameter that defines the ratio of infectious virions to cells in a culture. The establishment of the best MOI is crucial to characterize a lytic bacteriophage to be eventually employed for phage therapy. The optimal MOI value for ɸSaI_NFG_5581 and ɸSaI_NFG_5577 was defined as 0.1, which indicated that the phages could achieve a good bactericidal effect at a low concentration. Phage SaI_NFG_5581 slowed the host growth even after 24 h while ɸSaI_NFG_5577 can effectively control bacteria growth after 4 h, so their behavior is different.

In laboratory conditions, by using nutritive media culture, the development of resistance of bacteria against phages is expected. Moreover, the host employed in this research had a high growth rate and it was expected that a single phage exposure would not be able to kill the whole bacterial population. Nevertheless, bacteria total counts treated with phages never reached the control values (non-phage-treated bacterial counts), after 24 h, underlining phages can alter the fitness of bacteria and control their multiplication. Similar growth trends were obtained also by Kangachar et al. (2024) while studying phages against *E. coli*.

Since phages are often employed in mixed formulations (cocktails), the authors treated *S. Infantis* with both phages applied sequentially and together as a mix. Phages ɸSaI_NFG_5581 and ɸSaI_NFG_5577 did not exert a high synergistic activity and no significant benefit from using them together or after a sequential inoculation was observed, although the cocktail formulation resulted in a slightly more efficient bacteriolytic activity.

These data strengthen the concept that, other than phage characterization, the optimization of the experimental factors (MOI, phage application, temperature, type of matrix) is crucial to identify the most efficient protocol to establish an *ad hoc* therapy for each phage/host. These hypotheses have also been brought to light by other researchers (Pereira et al., 2017; Sun et al., 2022; Khan et al., 2024).

Moreover, from the perspective of tailored phage therapy, the isolation of phages in the same environment for future application could be a winning strategy, as proved by Sevilla-Navarro et al. (2018) with the application of auto-phages in laying hens. Sevilla-Navarro et al. (2024) also applied *S. Infantis* bacteriophages as disinfectants on poultry farms and the promising results obtained stress how phage potential could be exploited as an alternative control strategy against pathogens. Host range revealed that both phages in this work were able to lyse *S. Infantis* serovar, confirming their specificity. This feature is one of the major advantages of using bacteriophages: their high specificity is the guarantee of an accurate targeting, for prevention or treatment methods. At the same time, if target flexibility is needed and the treatment is required for reducing different bacterial species or strains, it is still possible to use some techniques, such as engineering methods, to broaden the phage host spectrum (Ando et al., 2015; Mahichi et al., 2009; Yoichi et al., 2005), or to mix phages with different host ranges.

The other side of the coin is that not all phages are suitable for controlling pathogens. For safety reasons, phages that carry antibiotic-resistance genes, lysogenic genes, or pathogenic genes must be avoided as biological control agents. Research has demonstrated that bacteriophages with genes for certain virulence factors can be harmful once they infect host bacterial cells, posing risks to human health. Additionally, bacteriophages could facilitate the horizontal transfer of drug-resistant genes among bacteria (Loc-Carrillo and Abedon, 2011; Philipson et al., 2018). Therefore, genetic analysis is essential to determine the potential application value of new phage isolates.

According to the International Committee on Taxonomy of Viruses (ICTV), ɸSaI_NFG_5581 can be ascribed to the class of Caudoviricetes, family Demerecviridae, and ɸSaI_NFG_5577 to the same class but family Guernseyvirinae. The phylogenetic tree based on the whole genome sequences and the DNA polymerase sequences showed that ɸSaI_NFG_5581 belongs to the *Tequintavirus* species and ɸSaI_NFG_5577 belongs to the *Cornellvirus* species.

In this study, genetic examinations were performed for both phages. The genome length of ɸSaI_NFG_5577 is 42,481 bp, which is similar to other *Salmonella* phages such as ɸvB_SenS_SE1 (40,987 bp, MK479295.1), TS6 (41,515 bp, MK214385.1), vB_SenTO17 (41,658 bp, MT012729.1), PSDA-2 (40,062 bp, MW725301.1); Shemara (44,342 bp, MN070121.2), FSL SP-031 (42,215 bp, NC_021775.1), VSiA (43,110 bp, MN393079.1), VsiP (42,865 bp, MH424444.1), GRNsp27 (41,187 bp, NC_069147.1), and St162 (42,701 bp, NC_069146). On the contrary, ɸSaI_NFG_5581 showed a longer genome sequence (112,970 bp), which was like other phages with similar genetic features, including Spc35 (118,351 bp, NC_015269.1), ECOP18 (111,345 bp, MT080103.1), BB1 (110,099 bp, MT843274.1), vB_Sen_I1 (112,111 bp, MT233524), and vB_SalS_SA001 (110,965, MN445182.1).

SaI_NFG_5577 phage genome contains 72 ORFs, of which 35 have annotated functions, and the rest are hypothetical proteins. SaI_NFG_5581 phage genome contains 209 ORFs, 77 of them have annotated functions, while the rest are hypothetical proteins. Some of them are predicted to be holin, spanin, and endolysins, which are proteins involved in invasion processes and lysis of the host (Fischetti, 2018). Holin is a small polar transmembrane protein produced in the later stages of phage infection. It creates non-specific damage to the bacterial cytoplasmic membrane, forming a stable transmembrane pore. This pore allows endolysin to access the peptidoglycan layer of the cell wall, where it can then perform its bacteriolytic function (Krupovic and Bamford, 2008; Young et al., 2000). The genomes of two phages isolated in this research contain several endonucleases. The absence of these enzymes could lead to a superspreader phenotype and the dispersal of intact plasmid DNA (Keen et al., 2017), while the presence of these proteins is desirable for phage therapy implications: endonucleases degrade host DNA and avoid gene transfer.

In the SaI_NFG_5577 phage genome, no tRNA genes were detected, indicating that the isolated phages possibly utilize the host tRNA machinery to synthesize proteins (Chen et al., 2021). On the contrary, in ɸSaI_NFG_5581 a tRNA cluster of 19 tRNA genes was predicted. Even though the presence of tRNAs in phages has not been completely understood, they may contribute to a higher virulence of the phage. Bailly-Bechet et al. (2007) demonstrated with a master equation model that tRNAs are the only translation-related genes commonly found in phages but they are not selected to help phage integration into prokaryotic chromosomes. Bailly-Bechet et al. (2007) also studied the distribution of the tRNA in phage sequences and discovered that virulent phages have more tRNAs than temperate phages, suggesting that tRNA acquisition contributes to increased virulence. Their presence can also favor the replication cycle, probably providing an advantage for expanding their host range and allowing bigger phages to increase their progeny (Lomeli-Ortega and Balcázar, 2024). The phylogenetic analyses based on the DNA polymerase sequences were useful in giving a taxonomy position of the two assessed phages. Polymerases are crucial to viral replication and may have a critical role in shaping the evolutionary history and fitness of the viruses that carry them. This approach is important to study the evolutionary relationships among different organisms by examining conserved genes, such as DNA polymerase genes (Doublie et al., 1998; Wommack et al., 2015; Nasko et al., 2018).

## 5 Conclusion

Two novels *S. Infantis* phages were isolated and characterized in this study. These phages belong to different families, are strictly lytic against *S. Infantis*, and have good biological features, which allow them to be considered promising candidates for phage therapy. Moreover, genomic analysis showed the absence of virulence factors, antibiotic resistance, and lysogeny genes in their genomes.

*S. Infantis* is a serovar currently highly prevalent in broiler chicken production, making the identification and characterization of bacteriophages against this serovar highly advantageous. Phages have demonstrated their potential as a complementary measure for its control on farms, and establishing a safe and effective phage collection represents a promising strategy for managing this pathogen. The uniqueness of phages also lies in their host specificity and, employing phages in the same environment of isolation could be a key factor for the fight against persistent pathogens.

Despite the promising findings, further characterization before *in vivo* trials is necessary to establish optimized parameters and to gain more insights into the suitability of those phages, alone and in a cocktail solution, to be used in therapy.

## Data Availability

The datasets presented in this study can be found in online repositories. The names of the repository/repositories and accession number(s) can be found in the article/Supplementary material.

## Footnotes

^1^https://genpat.izs.it/cmdbuild/ui/#login, accessed on 05 August 2024
