# Supplementary figures and images for: In vitro characterization and genome sequencing of two novel lytic phages against Salmonella Infantis isolated from poultry feces
Source: Front Microbiol. 2024 Dec 5;15:1479700. doi: 10.3389/fmicb.2024.1479700 (PMC11655500; doi:10.3389/fmicb.2024.1479700)

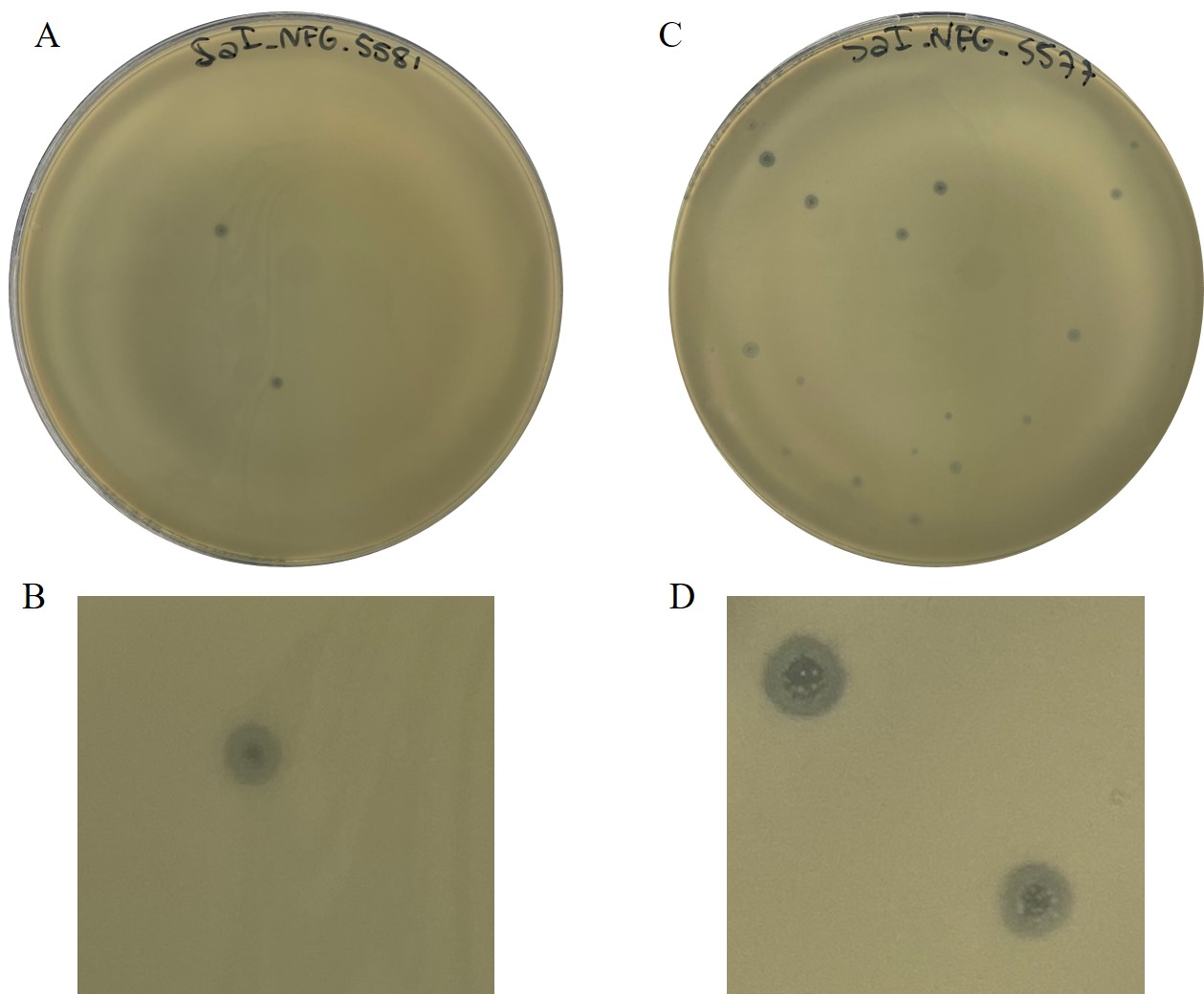

Supplement: Supplementary file 1 [file Image_1.jpeg]
